# Knowledge, Attitudes, and Practices Regarding Biosimilars Among Pharmacists and Physicians in Saudi Arabia: A Scoping Review

**DOI:** 10.7759/cureus.93099

**Published:** 2025-09-24

**Authors:** Abdulrhman Alanizi, Reema Alotaibi, Ranad Babalghaith, Abdullah Alzahrani, Reem Qubaiban, Ahmed Hattan, Mohammed Alnuhait, Alaa Al Sharif

**Affiliations:** 1 Pharmaceutical Care Services, King Abdullah bin Abdulaziz University Hospital, Riyadh, SAU; 2 Pharmacology, College of Pharmacy, Princess Nourah Bint Abdulrahman University, Riyadh, SAU; 3 Pharmaceutical Care Services, King Faisal Specialist Hospital and Research Centre, Riyadh, SAU; 4 Pharmaceutical Care Services, King Saud Medical City, Riyadh , SAU; 5 Pharmaceutical Practices, College of Pharmacy, Umm Al-Qura University, Makkah, SAU

**Keywords:** attitudes, biosimilars, healthcare providers, knowledge, knowledge attitude practice (kap), pharmacists, physicians, practices, saudi arabia, scoping review

## Abstract

Biosimilars were introduced in medical practice in recent years. The introduction of biosimilars aimed to provide more cost-effective treatment and improve patients' access to effective treatment. These drugs have recently been approved for use in Saudi Arabia. To optimize utilization of such medications, healthcare providers should be knowledgeable and have a positive perspective toward biosimilars. This scoping review aims to explore the knowledge, attitudes, and practices (KAP) of pharmacists and physicians in Saudi Arabia regarding biosimilars. The scoping review followed the Preferred Reporting Items for Systematic Reviews and Meta-Analyses (PRISMA) protocol. The databases searched for this review included PubMed, MEDLINE (Medical Literature Analysis and Retrieval System Online), EMBASE (Excerpta Medica Database), Scopus, and Google Scholar, covering relevant studies published between 2015 and 2024. A total of 59 studies were identified, and 10 met the inclusion criteria. The findings from the review revealed that approximately 48%-70% showed adequate knowledge of biosimilars. More than 60% showed a positive attitude among practitioners. Adoption was associated with familiarity with clinical evidence, specialty (e.g., oncology, gastroenterology, endocrinology), and clarity of regulatory guidance. The review identified gaps in regulatory knowledge and highlighted that targeted educational programs and policy interventions are needed to improve biosimilar adoption in clinical practice in Saudi Arabia.

## Introduction and background

Biosimilar medications, sometimes referred to as biosimilars, are competing versions of numerous biological drugs that are created by different firms using different cell lines [1]. While these entities are not completely identical, they do exhibit structural and functional similarities that should be evident in their effectiveness, clinical characteristics, and safety [2,3].

The introduction of biologics has revolutionized the treatment and prognosis of diseases in various specialties [4], though at the cost of higher direct costs [5]. Biosimilars have been introduced as a result of the patentability of biologics that are commonly prescribed [6]. The primary reasons for the need to bring new technologies into the healthcare system were to lower the price of biologics, improve patient access to medicines, and promote competition among businesses looking to enter the biotechnology sector [7]. 

Currently, these novel pharmaceuticals have emerged as a significant component of pharmacotherapies and are widely employed by numerous healthcare professionals for the management of various medical conditions, including rheumatoid arthritis, cancer, Crohn's disease, colitis, diabetes mellitus, osteoporosis, anemia, immunologic disorders, and other ailments. These therapeutic interventions are suitable for both adult and pediatric populations [8].

The use of biosimilars has increased in Saudi Arabia. For a biosimilar to be successfully integrated into clinical practice, healthcare professionals' knowledge, attitude, and practice (KAP), particularly that of physicians and pharmacists, are essential. The Saudi Food and Drug Authority (SFDA) has a crucial role in overseeing the regulation of biosimilars in Saudi Arabia. It establishes rules for the registration, quality assurance, and management of these biosimilars. The biosimilar market in Saudi Arabia is growing due to the presence of health transformation initiatives, the presence of regulations on using biosimilars in Saudi Arabia, and the implementation of value-based concepts. The goal of using such therapeutic agents is to enhance accessibility and reduce healthcare expenses [9], since the use of biosimilar alternatives may be cost-effective in terms of being a cheaper alternative and saving lives. Currently, there is reluctance in resorting to the practice of biosimilar use, and there exists a knowledge gap regarding the policies, safety, and use of biosimilars among various healthcare professionals in Saudi Arabia. This scoping review seeks to have a deep insight into the use and perception of biosimilars by pharmacists and physicians in Saudi Arabia, which significantly impacts the consumption of biosimilars.

The literature suggests considerable heterogeneity in the knowledge and practices related to biosimilars among healthcare professionals in Saudi Arabia. Researchers have identified a lack of knowledge vis-à-vis policy regulations and legality, safety profile, and the approval process of biosimilars in Saudi healthcare settings [10]. Another study investigated the role of education in promoting the adoption rate of biosimilars or increased practice of biosimilars by imparting knowledge among healthcare professionals [11]. These studies, though, offer an insight into the level of knowledge and practice of biosimilars in Saudi Arabia, but they may be narrow in scope due to limited context and a smaller sample size. Further investigation aimed at a larger sample size and wider context is needed to offer an inclusive and wider picture of the true landscape of knowledge about biosimilars.

In addition to knowledge about regulations and policies regarding biosimilar usage, cost-efficiency, safety profile, and perception about the effectiveness of biosimilars are some other factors influencing the attitude towards biosimilars. A study has reported a positive attitude towards the practice and adoption of biosimilars because of their potential for better health outcomes and more access to essential medicine [12]. However, there are concerns about the equivalence of effectiveness of biosimilars and their potential risk of adverse effects, leading to a more reserved and careful approach towards using biosimilars [12]. A study has offered a broad spectrum of opinions of healthcare providers and their attitudes towards the use of biosimilars. However, the literature lacks a holistic approach since it exclusively targets pharmacists, and the rest of the healthcare stakeholders are left out of consideration. Further investigation into the contextual elements that affect attitudes is essential [12]. 

The execution of efficient and pragmatic policy measures is crucial to facilitate the greater adoption of biosimilars in Saudi Arabia [13]. It is significant for regulatory bodies to remain committed to offering clear directions about the authorization and institution of biosimilars while also addressing concerns expressed by healthcare givers and patients [13]. Educational initiatives targeted at healthcare givers and patients have the potential to improve awareness and understanding of biosimilars, hence improving the process of making well-informed decisions. 

Having a detailed understanding of the KAP vis-à-vis biosimilars is essential in order to optimize its application and improve patient accessibility to necessary treatments in Saudi Arabia.

Although the current literature provides useful insights, there are several gaps that need to be addressed. Firstly, it is essential to conduct studies with larger, more inclusive, and diverse samples to ensure the generalizability of outcomes. Saudi Arabia has the capability to leverage the benefits of biosimilars in healthcare delivery and patient outcomes through the identification and resolution of knowledge gaps and concerns, and the implementation of specific regulations and educational efforts. 

## Review

Methods

Protocol 

The articles meeting the aim of this review were selected according to the Preferred Reporting Items for Systematic Reviews and Meta-Analyses (PRISMA) and its version, the PRISMA Protocols (PRISMA-P) [14]. 

Google Scholar was used as a search engine, while PubMed, Medical Literature Analysis and Retrieval System Online (MEDLINE), Excerpta Medica database (EMBASE), and Scopus were used to identify relevant articles. The studies published after 2015 were selected since the first official biosimilar regulation guidance in Saudi Arabia was published in 2016 [15].

Eligibility Criteria 

The studies examining the KAP of biosimilar usage by physicians and pharmacists were included. The studies cover the period from 2015 to 2024. Observational designs, primarily cross-sectional surveys, were eligible. Grey literature, non-peer-reviewed sources, editorials, and studies not reporting KAP outcomes were excluded.

Search Strategy 

The strategy of systematic search was meticulously designed to retrieve all the publications examining the KAP regarding biosimilars among healthcare providers in Saudi Arabia. The search strategy underwent peer review by four independent reviewers. 

The search followed established protocol and employed a time range filter, as this carried out a methodical search of various research databases. Database searches included PubMed, MEDLINE, EMBASE, and Scopus. The goal of following the protocols was to offer the maximum degree of transparency. 

The objective of the data collection process was to use an extensive array of relevant search phrases, which included synonyms and combinations. A number of search methods were used, such as phrasal search and keyword search. Key words used included [KNOWLEDGE], [ATTITUDE], [PRACTICE], [BIOSIMILAR], [MEDICINE], [DRUGS], [PHYSICIAN], [PHARMACIST], [HEALTHCARE], and [SAUDI ARABIA] coupled with the Boolean operators AND and OR as (knowledge OR attitude OR practice) AND (KSA OR Kingdom of Saudi Arabia OR Saudi Arabia) AND (biosimilar) AND (drug OR medicine). 

While using PubMed and MEDLINE databases, MeSH terms indexing was extensively used. Each key term within the research question, including "knowledge," "KSA," and "biosimilar," was mapped to relevant MeSH terms. For example, "knowledge" may be mapped to the MeSH term "Knowledge," "KSA" to "Kingdom of Saudi Arabia," and "biosimilar" to "Biosimilar." This mapping strategy ensures accuracy and consistency in retrieving related literature in different databases. Additionally, MeSH terms were integrated into the search strings alongside other keywords using Boolean operators to construct comprehensive search queries as ("knowledge"[Mesh] OR "attitude"[Mesh] OR "practice*") AND ("Saudi Arabia"[Mesh] OR "KSA" OR "Kingdom of Saudi Arabia") AND "biosimilar"[Mesh] AND ("drug" OR "drugs" OR "medicine" OR "medicines"). 

Study Selection 

The articles that met the eligibility criteria underwent a comprehensive and rigorous selection process. The titles and abstracts of the articles were screened for inclusion of studies. Following a preliminary screening of the title and abstract, the selected studies underwent a comprehensive evaluation of the whole text. During this phase, a complete analysis of the content of each paper in order to determine its compliance with all the inclusion criteria was conducted. The entire study selection process, including both initial screening and full-text evaluation, was rigorously peer-reviewed by four reviewers. 

Data Charting 

The process of data charting was started after the selection of studies that fell under the eligibility criteria. Table 1 includes all relevant data according to the outcome of classifications following screening of the article. Following data extraction, a narrative thematic synthesis was conducted to categorize findings into four domains: KAP and adoption drivers.

**Table 1:** Charted data from the selected studies KSA: Kingdom of Saudi Arabia; PK: pharmacokinetic

Study ID	Sample	Design	Outcome
Qahtani et al. (2024) [16]	Healthcare professionals	Cross-sectional study	Female pharmacists demonstrated higher intentions toward biosimilar compared to male pharmacists (35.5% vs. 28.1%)
Alahmari et al. (2021) [17]	Pharmacist	Cross-sectional study	A non-significant relationship between male and female pharmacists vis-à-vis the use of biosimilar medications in the KSA healthcare setting. Significant difference regarding familiarity with the need for preclinical and clinical evidence: 69.59% of pharmacists had correct knowledge about biosimilar drugs, and 60.5% had a positive attitude towards the use of biosimilars.
Almalki et al. (2020) [10]	Physicians	Cross-sectional study	Most physicians (84.8%) are convinced that the research regarding PK similarities between reference drugs and biosimilars is essential to be conducted for knowledge about efficacy and safety. Approximately 84.8% of the physicians showed interest in international and KSA clinical practice guidelines and the practice of biosimilars.
Awada et al. (2023) [18]	Pharmacists	Cross-sectional study	The pharmacists showed moderate to little knowledge regarding biosimilars. Most pharmacists observed that generic drugs could be substituted if the brand is not available. Pharmacists underscored the need to include biosimilars in the workshops and education program.
Iqbal et al. (2020) [19]	Pharmacists	Cross-sectional study	Most of the pharmacists (>60%) had a positive attitude towards the use of biosimilar drugs.
Ismail et al., (2023) [20]		Guidelines	Knowledge gaps need to be addressed by educating healthcare providers and patient education for successful transition to biosimilar drugs and policy development.
Omair et al. (2022) [21]	Physicians	Cross-sectional study	A total of 48.25% of physicians demonstrated adequate knowledge of biosimilars. Additionally, 61.5% reported that the available evidence was adequate to support biosimilar approval for the studied indication. The concept of the 'totality of evidence' was well understood by 37.1% of respondents. Furthermore, 30.07% had previously used biosimilars in their clinical practice.

Results 

Study Characteristics 

**Figure 1:**
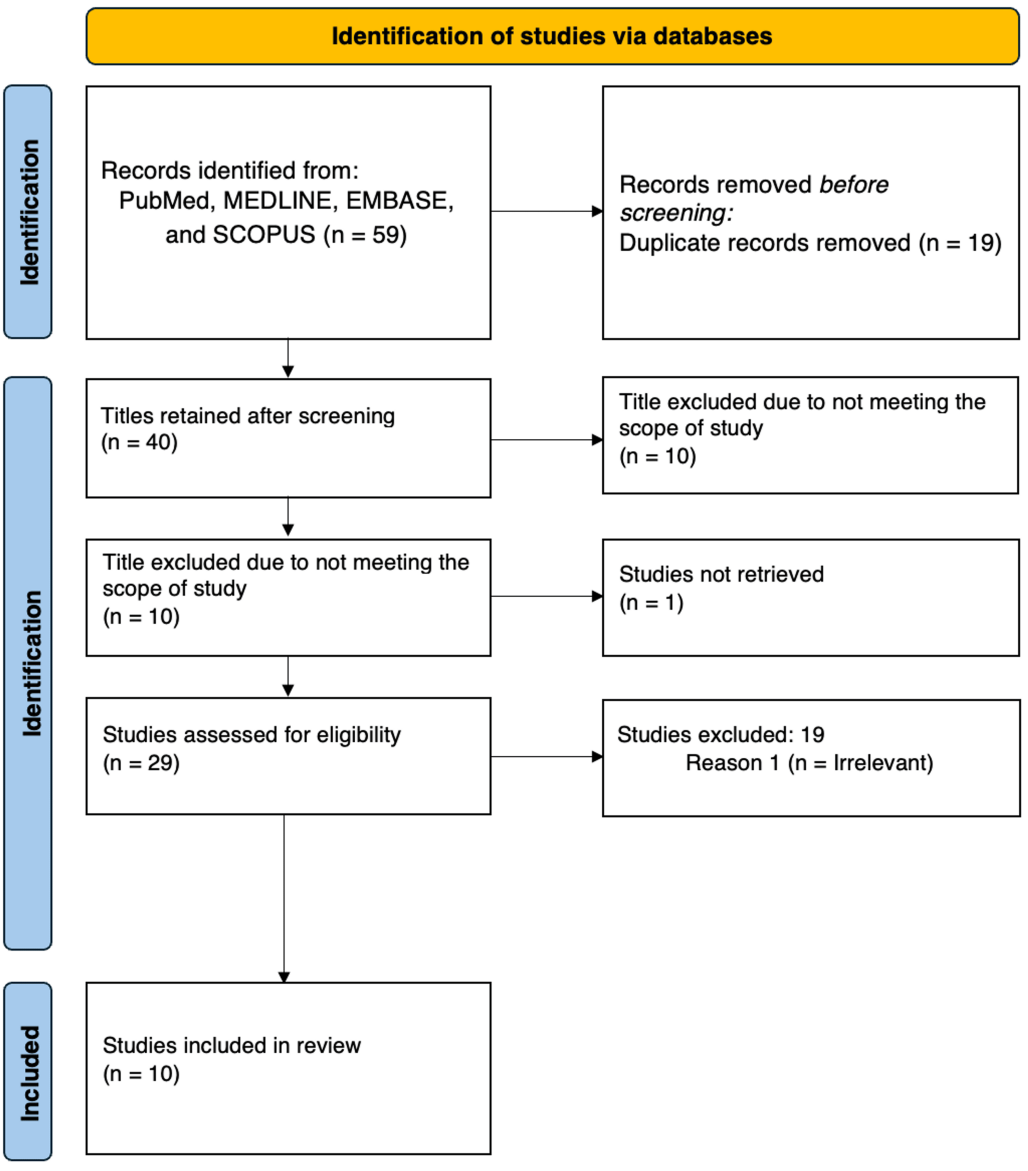
PRISMA flow diagram displaying data selection and charting process PRISMA: Preferred Reporting Items for Systematic Reviews and Meta-Analyses; MEDLINE: Medical Literature Analysis and Retrieval System Online; EMBASE: Excerpta Medica database

A total of 59 studies were found for the screening, and 10 fit the inclusion criteria for the purpose of this scoping review (Figure 1). Most of the studies included were cross-sectional surveys conducted in the Kingdom of Saudi Arabia (KSA). Three studies targeted pharmacists, two studies surveyed physicians, and one study included both the physicians and the pharmacists. The Sample, Phenomenon of Interest, Design, Evaluation, Research type (SPIDER) framework was used to structure the research question, as it is suited to qualitative evidence synthesis and studies focusing on perceptions and practices, such as KAP (Table 2) [22]. 

**Table 2:** SPIDER framework for key elements of the research question SPIDER: Sample, Phenomenon of Interest, Design, Evaluation, Research type [22]

Key elements	Attributes
Sample	Physicians, pharmacists
Phenomenon of interest	Knowledge, attitude, and practice regarding biosimilars among physicians and pharmacists in Saudi Arabia
Design	Scoping review
Evaluation	Level of knowledge, attitude, practice regarding biosimilar, and determination of factors influencing these phenomena
Research	Qualitative

KAP Adoption Drivers

Several studies revealed the level of knowledge among healthcare professionals, namely physicians and pharmacists, vis-à-vis biosimilars in Saudi Arabia. One of the studies underscored a gap in the intention of use of biosimilars between female and male professionals and showed a higher intention among females (35.5%) compared to males (28.1%) [16]. Likewise, two studies found that physicians, particularly in specialties like oncology, gastroenterology, and endocrinology, emphasized the importance of research on pharmacokinetic similarities between biosimilar and reference drugs, with approximately 84.8% [10,12]. 

The attitude regarding biosimilar medicines among healthcare professionals, particularly pharmacists, varied across studies. One study observed that the majority of pharmacists (60.5%) had a positive attitude towards the use of biosimilars; similarly, Iqbal (2020) revealed that around 60% of pharmacists surveyed showed a positive attitude [18, 19]. With regard to physicians, another study reported a significant share of physicians (48.2%) demonstrating adequate knowledge on biosimilars, with more than 60% reporting enough evidence for the approval of biosimilars in studied indications [21]. 

In addition to KAP regarding biosimilars, factors affecting the use of biosimilars were also examined in this review. These factors included provider demographics, medical specialty, familiarity with clinical and regulatory evidence, participation in educational programs, and perceptions of guideline and regulatory clarity.

Multiple factors were identified as influencing adoption. A study found that both gender and length of service influenced intention, with longer professional experience linked to higher acceptance [16]. Physicians in specialties like oncology, gastroenterology, and endocrinology expressed greater interest in biosimilars compared to generalists and community pharmacists [10]. Also, another study investigated the utilization of biosimilars in rheumatoid arthritis, which is well-received and practiced in KSA [23]. Familiarity with preclinical and clinical evidence was associated with more positive attitudes, particularly among pharmacists [17]. Several studies emphasized the need for workshops and continuing medical education (CME) programs to improve understanding, with pharmacists noting biosimilars could be used as alternatives if supported by physician approval [18]. Harmonized guidelines, pharmacovigilance, and patient education were repeatedly cited as essential to strengthen confidence and enable wider adoption [20, 21, 24].

Discussion 

The results from the scoping review offer an important insight into the KAP and factors affecting the application and use of biosimilars among healthcare professionals in KSA. To our knowledge, this study is the first of its kind that gives a review of the KAP of biosimilars and factors influencing their adoption among healthcare providers in Saudi Arabia. 

Numerous physicians and pharmacists showed a positive attitude towards biosimilars, which is in line with studies from Russia [25] and the USA [26]; however, the proportion in KSA is less than that in other regions. Pharmacists, compared to physicians, had less knowledge about biosimilars and less positive attitudes. This finding stands in contrast with some other academic investigations, which documented higher knowledge regarding biosimilars among pharmacists [27, 28]. One study explored the gender aspect of adoption intention among healthcare providers. They reported that there was a wide disparity regarding the intention of biosimilar adoption and prescribing practice in healthcare professionals [16]. This conclusion complies with the findings from other studies from developed regions, Europe, and the USA, which validate the role of socio-cultural aspects in the adoption intention and adoption rate of biosimilars [29]. The variability in perception and attitude regarding biosimilars underlines the need to take contextual elements into account to help develop policies to encourage the practice of biosimilars. 

Another critical aspect influencing the perception and use of biosimilars is the degree of familiarity with evidence regarding their usage and efficacy, the extent of awareness of biosimilars, and the type of healthcare profession one works in. According to a previous study that was carried out in Europe, evidence-oriented and specialty-based education proved to be of great importance, as they strengthened the prescribing practice of biosimilars [30]. The result of implementing educational programs and training workshops will be improved comprehension about the importance of generic biosimilars and favorable attitudes towards them by healthcare practitioners [31]. 

Besides individual features, system-level differences also influence the practical use of biosimilars. Saudi Arabia has made notable progress in modernizing its healthcare system, and policies have been introduced to support the prescription and application of biosimilar medications. However, biosimilar uptake remains slower than in Europe and the United States, which may be attributed to the nature and design of the Saudi healthcare system, including its more decentralized structure and varied institutional pathways. In contrast, Europe and the United States have more centralized, guideline-driven systems that facilitate uniform implementation of biosimilar policies [32, 33].

The specific analyses highlight an ordinary need for personalized policy and regulatory transformations regarding the use of biosimilars. These changes should be tailored to effectively address the unique challenges and opportunities within Saudi Arabian health systems. Policies and practices in developed nations can be translated into the Saudi context to help overcome hurdles and encourage the use of biosimilars by healthcare stakeholders. 

Future research undertakings should focus on longitudinal analyses to observe evolving practice and attitudes towards biosimilars among healthcare givers in KSA. The market of biosimilars is still nascent in Saudi Arabia; therefore, there is a lack of studies on the practice of biosimilars among healthcare providers in Saudi Arabia. However, this scoping review has intended to cover all the possible studies conducted in Saudi Arabia to give an insight into the biosimilar landscape in Saudi Arabia. This review included a small number of studies and focused on studies conducted in Saudi Arabia, which may limit the generalizability of findings to other healthcare systems in the Gulf Cooperation Council (GCC) region. To support wider adoption of biosimilars in Saudi Arabia, several actionable steps are recommended. First, the SFDA should establish clear national guidelines on interchangeability and substitution, as regulatory ambiguity has been shown to reduce prescriber confidence [34]. Second, CME should be implemented for pharmacists and early-career physicians, since a lack of structured training is associated with lower knowledge and uptake [18, 10]. Third, incorporating biosimilar-focused content into pharmacy and medical curricula could build early foundational knowledge and positive attitudes [35]. Finally, national studies are needed to investigate barriers and facilitators influencing biosimilar use, as addressing such context-specific factors can enhance adoption. These steps could help close current knowledge gaps and accelerate the safe, cost-effective integration of biosimilars into Saudi healthcare practice.

## Conclusions

This review highlights the KAP of healthcare providers in Saudi Arabia toward biosimilars. Physicians demonstrated more positive attitudes compared to pharmacists, and female providers showed greater willingness to use biosimilars than males. Bridging knowledge gaps through targeted education and training programs is crucial to encourage the adoption of biosimilars. Priority efforts should begin with regulatory harmonization to establish clear national guidelines, followed by structured education and training initiatives and sustained stakeholder engagement to ensure coordinated implementation.
